# Open-access network science: Investigating phonological similarity networks based on the SUBTLEX-US lexicon

**DOI:** 10.3758/s13428-025-02610-9

**Published:** 2025-02-18

**Authors:** John Alderete, Sarbjot Mann, Paul Tupper

**Affiliations:** Department of Linguistics, Simon Fraser University, 8888 University Drive, Burnaby, BC V5A 1S6 Canada

**Keywords:** Network science, Phonology, Phonological similarity networks, Degree, Data validation, SUBTLEX-US English

## Abstract

Network science tools are becoming increasingly important to psycholinguistics, but few open-access data sets exist for exploring network properties of even well-studied languages like English. We constructed several phonological similarity networks (neighbors differ in exactly one consonant or vowel phoneme) using words from a lexicon based on the SUBTLEX-US English corpus, distinguishing networks by size and word representation (i.e., lemma vs. word form). The resulting networks are shown to exhibit many familiar characteristics, including small-world properties, broad degree distributions, and robustness to node removal, regardless of network size and word representation. We also validated the SUBTLEX phonological networks by showing that they exhibit contrasts in degree and clustering coefficient comparable to the same contrasts found in prior studies and exhibit familiar trends after extraction of a backbone network of nodes important to network centrality. The data release (https://github.com/aldo-git-bit/phonological-similarity-networks-SUBTLEX) includes 17 adjacency lists that can be further explored using the networkX package in Python, a package of files for building new adjacency lists from scratch, and several scripts that allow users to analyze and extend these results.

## Introduction

Over the past 25 years, network science has led to significant progress in psycholinguistics. It has given the field a range of predictive measures for analyzing behavioral data, such as degree (a.k.a., neighborhood density) and clustering coefficient (for review, see Vitevitch and Castro (2015)). These measures and the mathematical methods developed for them enable graph-theoretic analyses of entire lexicons, not just the fragments of lexicons. While network models are not models of psycholinguistic behavior, they capture facts of relevance for these models, and in doing so, provide new insight into language processing in the mental lexicon (Chan & Vitevitch, 2009, 2010; Siew & Vitevitch, 2016). For example, phonological networks based on form resemblance have high assortative mixing by degree, meaning that high-degree nodes tend to be linked to each other, and likewise for low-degree nodes. This mixing pattern accounts for some of the robustness of the network to node removal, and it has natural interpretations in terms of the gradual degradation of word retrieval (Arbesman et al., 2010; Vitevitch et al., 2014). In addition to the role of degree and clustering coefficient in early work on comprehension and production (Dell & Gordon, 2003; Munson & Solomon, 2004; Vitevitch, 1997, 2002), recent research has investigated new measures and data structures in the dynamics of learning and aging (Cosgrove et al., 2021; Fourtassi et al., 2020; Jones & Brandt, 2020), emergent community structure in language networks (Kovács et al., 2021; Siew, 2013; Tiv et al., 2020), and analysis of multiple networks in so-called multiplex network analysis (Castro et al., 2020; Stella, 2019). In sum, network science has room to grow in psycholinguistics.

Despite this progress, two problems currently exist that are constraining this growth. First, there are very few open-access resources that support the creation of network science measures for individual words or lexicons, even for well-studied languages like English, Dutch, and German. While some key measures (e.g., phonological and semantic neighbors) are documented in online repositories like the English Lexicon Project (Balota et al., 2007) and CLEARPOND (Marian et al., 2012), these repositories do not provide the data structures for deeper network analysis, such as adjacency or edge lists, that are required to generate from scratch other measures for individual words and global network properties for an entire lexicon. Perhaps because of the computational difficulty in producing these lists, prior research has not released the structures required for deeper analyses, requiring researchers to pay to use proprietary data repositories like Wolfram Data Repository to tackle new problems.

A second problem is that most psycholinguistic research using network science methods is skewed toward models with specific characteristics. To understand the types of networks studied and the digital resources underlying the research, we examined 47 network models surveyed in two recent review articles (Turnbull, 2021; Vitevitch & Castro, 2015). Because of their importance to form-encoding in psycholinguistics, most studies used phonological similarity networks, or networks of words where words are linked if they differ in the deletion, addition, or substitution of a single phoneme. Almost all these studies (42 or 89.36%) used the Hoosier Mental Lexicon (HML) as the basis for this kind of network. The HML is an electronic data set comprised of the 19,839 words from the 1964 Merriam-Webster Pocket Dictionary (Nusbaum et al., 1984). As many researchers familiar with it know, the HML is a valuable resource because word representations are expertly transcribed, and its size is a realistic approximation of the mental lexicons of university students commonly recruited for psycholinguistic experiments. That said, this data set is not publicly available, nor are the underlying data structures required to fully explore it. In addition, research that employs this network is limited to word lemmas because the entries of the HML are dictionary headwords, and so related word forms are not included (e.g., *play*, but not *plays* and *playing*). In sum, because of these resource limitations, this research is limited by the languages investigated, the type of word representation, and the size of the network.

To address this gap, we have developed and investigated a set of phonological similarity networks based on open-access principles. In particular, we have created 17 phonological networks from the SUBTLEX-US English corpus, a public data set of word forms compiled from movie and TV subtitles (Brysbaert & New, 2009). We chose this corpus because it is developed to a set of standards in psycholinguistics that has made it one of the most popular corpora for investigating psycholinguistic behavior, including open access, published frequency norms, part of speech tagging, and data validation on behaviors like lexical decision times (Brysbaert & New, 2009; Brysbaert et al., 2012, 2014). Following the methods used in (Shoemark et al., 2016), we generated phonological networks of different sizes that distinguish word forms and word lemmas extracted from the corpus in order to test the impact of these factors on network characteristics. We also examine below the mathematical properties of these networks and compare them with other documented networks (Arbesman et al., 2010; Castro & Vitevitch, 2023; Shoemark et al., 2016; Vitevitch, 2008) and validate the new networks by comparing their network properties to those of the HML from several prior experimental and mathematical studies. The resulting digital resources include the phonological networks encoded as adjacency lists, which, by representing all the phonological neighbors for each word, can be explored further with programming frameworks such as Python and R.

## Methods

### Phonetic transcription

We require a phonetic representation for each of the 74,286 words in SUBTLEX-US in order to build a phonological similarity network. To this end, we first converted a majority of word forms to IPA representations using the Python program *eng-to-ipa* (Mphilli et al., 2019). This program uses confirmed representations from the Carnegie Mellon University Pronouncing Dictionary (Weide, 2014), which provides phonetic transcriptions of North American English suitable for the SUBTLEX-US data set. However, many word forms (25,931, or roughly 35%) did not have an entry in the CMU dictionary, and so we require grapheme-to-phoneme conversion to include these data. This type of conversion is difficult in a language like English because of grapheme ambiguity and variability, irregular spellings, homophones, and many-to-one mappings, and thus the state-of-the-art employs neural network models like those used in machine translation to address these problems. We selected the grapheme-to-phoneme model developed in Ryan (2020) to construct the missing IPA representations. This model uses the OpenNMT open-source toolkit for neural machine translation (Klein et al., 2017) to train and test an encoder-decoder model that performs with high accuracy. It was also trained in part on the CMU data set we use here, making it suitable for our application.

To test the accuracy of the output forms, we sampled 1000 words of SUBTLEX-US three times and compared the neural model’s output with the CMU reference form when it was available. The neural model’s IPA output was identical to the CMU reference 90% of the time. Non-identical words also resembled the CMU reference forms to a considerable degree. The non-identical output forms had a string edit distance of 1.78 from the CMU reference form, meaning that they were typically only one or two phonemes off. Inspection of these forms showed that errors typically occurred in vowel representations and often involved variant pronunciations that are attested in some speech varieties, as in [dɪspɹuvən], cf. reference [dɪspɹuvɪn] for ‘disproven’. While the IPA representations are not perfect, we believe that they are sufficiently accurate to proceed with building networks.

### Building the phonological networks

We used the IPA representations of SUBTLEX-US word forms described above to construct several phonological similarity networks. In particular, we created an adjacency list (adjlist) for each network, which is, in essence, a list of lists in which each list begins with a word (a node in the network) that is followed by all its neighbors. In other words, the adjlist is a long data table in which each row starts with a word in the node network followed by all other phonologically related words linked to it. Following standard practice, phonological neighbors are words that differ in the substitution, addition, or deletion of a single phoneme (Luce & Pisoni, 1998).

To investigate the effect of size on network properties, we sampled from the complete networks to produce smaller ones while retaining the word frequency distributions of the larger corpus. This was achieved by using a technique of thresholding by word frequency, which effectively simulates building a lexicon from a smaller corpus by removing low-frequency words (Shoemark et al., 2016). The logic of thresholding is that smaller corpora would be more likely to contain a higher percentage of high frequency than a larger corpus. Thought of in acquisitional terms, small children will be exposed to less low-frequency words than adults because they experience fewer words, and there is a greater chance of them being high-frequency. Thus, by removing low-frequency words below some frequency cutoff, we approximate the composition of these corpora. Concretely, we created sub-networks with different orders of magnitude by randomly removing low-frequency words (i.e., words below the median word frequency) based on their frequencies in SUBTLEX-US. In addition, to compare networks based on SUBTLEX-US with the HML, we created networks that matched the size of the HML.

Nine of our phonological networks are built with word forms as nodes. We also created eight networks with word lemma nodes using the spaCy lemmatizer (spacy.io) to select appropriate words representing word lemmas. Again, using thresholding by word frequency, the networks differ in orders of magnitude, and we also include the entire SUBTLEX-US lemma list as well as one matched in size with the HML. Finally, because phonological similarity networks are blind to morphology, they will automatically include many inflected word forms as neighbors of a word base (e.g., *cat/cats*) in networks with word forms as nodes. Though these morphologically related forms may impact phonological processing, we opted to remove these effects because they conflate morphological and phonological relatedness. To this end, we built a special-purpose filter that removes edges between words that share the same stem and differ only in inflection. After experimenting with this filter, we found that it can have a real impact on node relationships in the word-form networks (it was not applied to the lemma networks because they lack the relevant word pairs). For example, 3851 edges (though no nodes) were removed from the word-form network with 19,839 nodes, or 19.41% of the logically possible edges.

## Results and discussion

### Descriptive statistics

We start with statistics that describe the gross structure of different components of our word form and lemma networks. Following Vitevitch (2008), we distinguish the giant component (i.e., the sub-network with the largest number of connected nodes), the nodes inside so-called lexical islands (all sub-networks other than the giant component with more than one node), and lexical hermit nodes with no neighbors (see the appendix for a glossary of all the technical terms we use). Table 1 gives the node counts (and percentages of all network nodes) for all 17 phonological similarity networks. The size of each network ranges from 1024 nodes and rises by orders of magnitude to the total count of all words in SUBTLEX-US. As expected, network density falls as the networks grow because the number of possible links (= *N/*(*N*−1)/2) grows much faster than actual links as the network sizes increase. These statistics and many other measures given below were calculated using methods from the networkX Python package (Hagberg et al., 2008).

**Table 1:** Counts of phonological network components and other descriptive statistics (parentheticals give the percentage of nodes in all three components: giant, island, and hermits)

	Size	Nodes	Links	Density	Nodes in giant	Nodes in islands	Hermit nodes	Islands
Word forms	2^10^	1024	1689	0.00323	555 (54.20)	100 (9.77)	369 (36.04)	35
	2^11^	2048	4107	0.00196	1066 (52.05)	205 (10.01)	777 (37.94)	78
	2^12^	4096	8907	0.00106	2035 (49.68)	423 (10.33)	1638 (39.99)	163
	2^13^	8192	20,204	0.00060	4020 (49.07)	833 (10.17)	3339 (40.76)	329
	2^14^	16,384	45,900	0.00034	7944 (48.49)	1673 (10.21)	6767 (41.30)	674
	2^15^	32,768	101,182	0.00019	14,949 (45.62)	3749 (11.44)	14,070 (42.94)	1496
	2^16^	65,536	217,156	0.00010	28,117 (42.90)	8837 (13.48)	28,582 (43.61)	3405
	All	74,286	256,542	0.00009	32,097 (43.21)	10,255 (13.80)	31,934 (42.99)	3936
	~ HML	19,839	57,676	0.00029	9482 (47.79)	2106 (10.62)	8251 (41.59)	841
Lemmas	2^10^	1024	1704	0.003253	529 (51.66)	106 (10.35)	389 (37.99)	43
	2^11^	2048	4108	0.00196	1028 (50.20)	217 (10.60)	803 (39.21)	82
	2^12^	4096	9902	0.001181	1998 (48.78)	448 (10.94)	1650 (40.28)	165
	2^13^	8192	22,514	0.000671	3839 (46.86)	858 (10.47)	3495 (42.66)	342
	2^14^	16,384	49,473	0.000369	7162 (43.71)	1807 (11.03)	7415 (45.26)	736
	2^15^	32,768	108,177	0.000202	13,134 (40.08)	4057 (12.38)	15,577 (47.54)	1636
	All	49,690	172,115	0.000139	19,172 (38.58)	7018 (14.12)	23,500 (47.29)	2782
	~ HML	19,839	62,455	0.000317	8485 (42.77)	2261 (11.40)	9093 (45.83)	917

These results reveal a difference between the Hoosier Mental Lexicon and a similarly sized SUBTLEX-US network in component sizes (Table 1, Lemmas, ~ HML). The giant component of the SUBTLEX-US network is somewhat larger, with 8485 nodes (42.77%) as opposed to 6508 nodes (33.65%) in the HML network.^1^ The uptake in the HML network is principally in the number of hermit nodes, 10,265 (53.08%), which greatly exceeds the giant component nodes in this network. This differs from the sized-matched SUBTLEX-US network, which only has a 3% difference in size between giant components and hermit nodes. While different from the HML network, the size of the SUBTLEX-US giant component is not particularly odd cross-linguistically. The average size of the giant component of phonological similarity networks from five linguistically distinct languages is 45.4% (Arbesman et al., 2010), which compares with the size of our HML-matched network. In our investigations below, we continue to use the lemma-based HML-matched network, despite the differences in component size, because there are no alternative networks that approach the HML giant component size.

### Backbone analysis

Given the difference in giant component size, we compared the HML and size-matched SUBTLEX-US networks with the statistical technique of backbone extraction. The creation of a so-called backbone network involves removing redundant links in a network to identify “important” nodes in the sense that they make important contributions to the centrality measures of a network (Neal, 2022). Vitevitch and Sale (2023) analyzed the phonological backbone of the HML network and found that words removed from the original giant component differed significantly from those that were not removed in several network science and psycholinguistic measures. We applied the same tools to a similarly sized SUBTLEX-US network to determine if there were comparable differences before and after backbone extraction.

We employed methods similar to those used in Vitevitch and Sale (2023). We used our own implementation of the L-Spar algorithm (Satuluri et al., 2011) to extract the backbone from the SUBTLEX-US network in order to preserve and investigate community structure. We first tried the same parameters published in Vitevitch and Sale (2023), but this model resulted in a backbone network with far too many nodes removed from the original giant component. After some experimentation, we found that raising the sparsity parameter *s* from 0.0 to 0.5 resulted in a more reasonable number of extracted nodes (though still higher than the HML analysis). We then examined the same set of network characteristics before and after backbone extraction and analyzed the network science (degree, clustering coefficient, closeness centrality) and psycholinguistic (word length, word frequency) measures of individual words.^2^

As shown in Table 2, the extracted backbone network follows most of the trends found for the HML. In particular, the backbone network had major drops in the number of edges and average degree in both the giant component and the larger network, and the network’s diameter, average shortest path length, and the number of communities rose with magnitudes comparable to those in the HML backbone. As mentioned above, our chosen parameters had the effect of removing many more words from the backbone giant component, and this probably accounts for the difference in the number of components and their range in size (since backbone networks are guaranteed to have the same number of isolates, a major decrease in the size of the giant component will increase the number of non-giant components and their sizes).

**Table 2:** Comparison of original and extracted backbone networks (GC = giant component)

	Original network	Backbone network
Number of nodes in the network	19,839	19,839
Number of edges in the network	57,676	20,640
Number of nodes in GC	9482 (47.8%)	7069 (35.6%)
Number of edges in GC	56,347 (97.7%)	16,087 (77.9%)
Average degree in the network	5.81	2.08
Average degree in GC	11.89	4.55
Network diameter	25	74
Average shortest path length	6.58	18.8393
Number of connected components	841	1219
Size of components (min.-max.)	2–19	2–53
Number of isolates	8251 (41.6%)	8251 (41.6%)
Average clustering coefficient	0.14	0.18
Number of communities in the network	9124 (*Q* = 0.72)	9539 (*Q* = 0.96)

Likewise, as shown in Table 3, we found most of the significant differences in word properties found in Vitevitch and Sale (2023). This table contrasts the two relevant word classes (i.e., words from the original giant component and retained in the backbone giant component versus words originally in the giant component but removed from the backbone giant component) and found significant differences in the same direction in all measures: log_10_ word frequency (*t*(1) = 10.38, *p* < 0.001), word length (*t*(1) = −47.82, *p* < 0.001), degree (*t*(1) = 40.29, *p* < 0.001), and closeness centrality (*t*(1) = 381.68, *p* < 0.001). The one salient difference between the SUBTLEX-US and HML networks is that Vitevitch and Sale (2023) did not find a significant difference in average clustering coefficient between these two word classes, but we did, with an increase in cluster coefficient in words removed from the giant component in the backbone network (*t*(1) = – 13.26, *p* < 0.001). This difference correlates with a modest increase in clustering coefficient in the larger backbone network (Table 2). We do not have an explanation for this difference, other than our backbone network has removed many more relatively important words from the original giant component than the HML backbone network (approximately eight times as many). With such a drastic drop in potential neighbors in the giant component, this may reduce the clustering coefficients of words in this component. If this is true, then this difference is a consequence of our model parameters (see above) and not a major difference between the HML and SUBTLEX-US networks.

**Table 3:** Characteristics of words in and out of GC after backbone analysis

	Original GC	GC of backbone	Orig. GC/Not Bb
Number of nodes	9482	7069	2413
Frequency (log_10_)	0.78 (0.75)	0.82 (0.78)	0.65 (0.64)
Word length (# phonemes)	4.31 (1.12)	4.02 (1.03)	5.13 (0.96)
Degree	11.89 (13.65)	13.27 (13.50)	4.56 (7.12)
Clustering coefficient	0.28 (0.24)	0.29 (0.22)	0.40 (0.40)
Closeness centrality	0.16 (0.03)	0.18 (0.03)	0.01 (0.01)

### Small-world properties

In networks with so-called small-world properties, nodes tend to be connected to each other with a small number of intermediaries. For example, the US air transportation network has 546 nodes (airports) and only 2791 links (one-stop flights), so the minimum number of connecting flights between two airports could be large theoretically. However, the average path length between any two nodes is 3.2 (data from OpenFlights.org). More formally, small world networks have small average shortest path lengths and high average clustering coefficients when compared to the average clustering coefficients expected in equivalent *Erdős-Rényi* graphs, which are graphs that have the same number of nodes and edges but the edges are randomly inserted between node pairs (Watts & Strogatz, 1998). We expect our phonological similarity networks to exhibit small-world properties because they have been found in the HML similarity network (Vitevitch, 2008) and even in networks based on pseudo-lexicons in which words were generated by drawing phonological segments at random from a fixed sound inventory (Gruenenfelder & Pisoni, 2009).

We follow standard practice in calculating these measures from the giant component of a network (Vitevitch, 2008) and report average shortest path length and clustering coefficients for all networks in Table 4. To address the potential for variation in the *Erdős-Rényi* networks, we created ten such networks for each network size and type and report the mean values of each set of ten networks here. We also report the Humphries–Gurney small-world-ness measure *S*, which is a function of average shortest path length and clustering coefficients in these tables, and another standard technique for evaluating small-world-ness (Humphries & Gurney, 2008). Humphries and Gurney assume that a network with an *S* greater than 3 has small-world properties and only applies tests of significance to networks with a lower *S.*

**Table 4:** Average shortest path length, clustering coefficients, and Humphries–Gurney small-world-ness S for SUBTLEX-US and matched Erdős-Rényi (ER) networks

	Lemmas						Word forms			
	ASPL	ASPL-ER	CC	CC-ER	HG *S*	ASPL	ASPL-ER	CC	CC-ER	HG *S*
2^10^	5.1040	3.6451	0.3257	0.0115	20.22	5.0927	3.7708	0.3168	0.0103	22.67
2^11^	4.9543	3.6235	0.3171	0.0074	31.43	5.0989	3.6936	0.3119	0.0069	32.59
2^12^	5.4690	3.6103	0.3072	0.0048	42.50	5.9242	3.7973	0.2903	0.0042	44.51
2^13^	6.1190	3.6554	0.2970	0.0030	59.62	6.3423	3.8852	0.2864	0.0023	75.43
2^14^	6.1079	3.6938	0.2912	0.0019	93.21	6.5761	3.9606	0.2764	0.0014	116.75
2^15^	6.2012	3.7135	0.2744	0.0012	135.65	6.7520	3.9870	0.2727	0.0009	182.38

It is clear from these results that all networks, regardless of size or word representation, have small-world properties. They all have low average shortest path lengths and high average clustering coefficients. The latter is an order of magnitude greater than the average clustering coefficients of random matched networks, but this is not the case with average shortest path length, as found in prior studies. For example, the network closest in size to the HML network, with 2^14^ nodes (lemma representations), has a logical limit of 16,382 intermediaries (i.e., the number of nodes minus two for the source and target nodes), but an average path length of 6.1 (or 5.1 intermediaries on average). Likewise, the average clustering coefficient, 0.2912, is 153 times larger than the same value for a random matched graph. Finally, all networks have a Humphries–Gurney *S* far exceeding the threshold of 3 and their *S* values grow with network size. These facts all support the conclusion that the SUBTLEX-US networks are like other phonological networks in having small world properties, and that network size and word representation do not affect this outcome.

### Degree distributions

Many studies have investigated the degree distributions of phonological networks to gain insight into network structure. For example, the breadth of degree distributions, or degree variability across the network, can be indicative of structural constraints in the network, like the limitations imposed by using a limited segment inventory to form words (see Vitevitch (2008) and Arbesman et al. (2010) for discussion). Below we examine the degree distributions in our SUBTLEX networks in order to compare them with known networks used in psycholinguistics.

Following Vitevitch (2008), we analyzed the degree distributions of the interconnected giant components. The facts about these distributions in Table 5 show that all phonological networks are sparse in the sense that average degree is low relative to network size. Further, the low heterogeneity parameter values (see appendix) indicate that, while the degree distributions may be broad, the distribution is weighted toward low-degree nodes.

**Table 5:** Basic information about the degree distribution and the giant components of phonological networks

	Nodes	Nodes_GC_	Edges	Assortativity	Heterogeneity	Degree_Average_	Degree_Max_	Degree_SD_
Word forms	1024	555	1623	0.59	0.17	5.85	23	4.53
	2048	1066	3974	0.65	0.12	7.46	35	6.18
	4096	2035	8636	0.66	0.11	8.49	43	7.41
	8192	4020	19,665	0.71	0.09	9.78	54	9.59
	16,384	7944	44,859	0.75	0.08	11.29	79	12.60
	32,768	14,949	98,809	0.77	0.07	13.22	112	16.16
	65,536	28,117	211,361	0.77	0.06	15.03	141	19.30
	74,286	32,097	249,823	0.77	0.06	15.57	151	20.36
	19,839	9482	56,347	0.76	0.07	11.89	89	13.65
Lemmas	1024	529	1638	0.63	0.16	6.19	23	4.99
	2048	1028	3964	0.66	0.12	7.71	36	6.40
	4096	1998	9601	0.68	0.09	9.61	49	8.45
	8192	3839	21,973	0.72	0.08	11.45	68	11.78
	16,384	7162	48,345	0.74	0.07	13.50	96	15.57
	32,768	13,134	105,639	0.76	0.06	16.09	135	20.24
	49,690	19,172	167,630	0.75	0.05	17.49	150	22.72
	19,839	8485	61,039	0.75	0.06	14.39	102	17.02

**Fig. 1:**
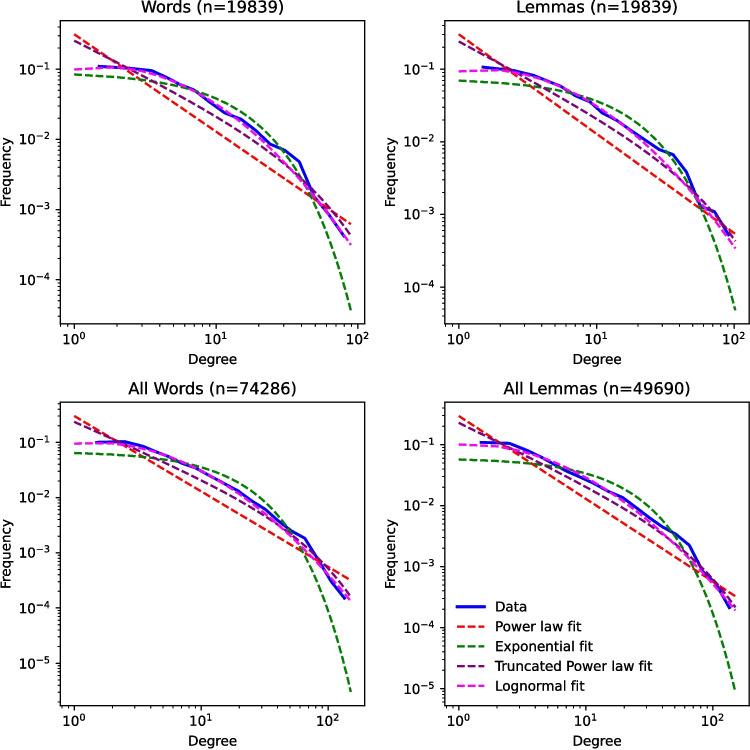
Curve fits of degree distributions

Degree distributions plot the count of nodes on the *y*-axis and degree of these nodes on the *x*-axis. These distributions are generally plotted in double logarithmic scale in order to resolve their shapes at different orders of magnitude. Prior research has attempted to flesh out the type of function that best characterizes these distributions. For example, Vitevitch (2008) argues that the degree distributions of the giant component of the Hoosier Mental Lexicon network are better characterized by an exponential function than a power-law function. Figure 1 shows the fit of the degree distributions with four classes of functions that have been parameterized to have the best fit within their class (fits with power-law functions were computed with the Python powerlaw toolkit from Alstott et al. (2014)). These fits, and Kolmogorov–Smirnov distances reported in Table 6, support two conclusions. First, the degree distributions from the SUBTLEX networks are curved for the whole range of degrees. This fact suggests that the distributions are not power-law or truncated power-law distributions, which should appear as straight lines on log–log scales. Second, the best fit functions in terms of their KS distance are exponential for networks with 4096 nodes or fewer and log-normal in networks with a greater number of nodes.

**Table 6:** Kolmogorov–Smirnov distance for fit of log–log degree distributions with four classes of functions

	Nodes	Nodes_GC_	Log-normal	Exponential	Power law	Truncated power law
Word forms	1024	555	0.0669	0.0359	0.2288	0.1396
	2048	1066	0.0604	0.0304	0.2336	0.3602
	4096	2035	0.0590	0.0320	0.2246	0.1329
	8192	4020	0.0414	0.0532	0.2138	0.1126
	16,384	7944	0.0306	0.0859	0.2167	0.1072
	32,768	14,949	0.0231	0.1022	0.2200	0.1054
	65,536	28,117	0.0173	0.1188	0.2126	0.0936
	74,286	32,097	0.0168	0.1266	0.2124	0.0919
	19,839	9482	0.0278	0.0906	0.2210	0.1097
Lemmas	1024	529	0.0621	0.0276	0.2236	0.1416
	2048	1028	0.0575	0.0308	0.2459	0.1622
	4096	1998	0.0606	0.0310	0.2356	0.1442
	8192	3839	0.0432	0.0670	0.2174	0.1114
	16,384	7162	0.0298	0.0965	0.2116	0.0974
	32,768	13,134	0.0240	0.1244	0.2060	0.0858
	49,690	19,172	0.0264	0.1482	0.1953	0.0716
	19,839	8485	0.0288	0.1078	0.2156	0.0998

The fact that some SUBTLEX networks have exponential distributions agrees with Vitevitch’s (2008) findings (though this work did not test for log-normal distributions) and Gruenenfelder and Pisoni (2009), which found similar results for artificially generated phonological networks. The fact that larger networks seem to follow a log-normal distribution is consistent with the findings of Siew and Vitevitch (2019) for degree distributions in a phonographic network that combines both phonological and orthographic similarity in a phonological network, though this type of distribution has only recently been explored.

### Robustness

Networks like transportation networks can be fragile in the sense that removing high-degree nodes, or hubs, can greatly impact the connectedness of other nodes. Language networks, on the other hand, have been shown to be robust to node removal (Arbesman et al., 2010; Vitevitch et al., 2023). This robustness has been demonstrated by comparing node removal at random and by descending degree, and then assessing the impact of the node removal on average path length and the size of the giant component. For example, Arbesman et al. showed that there is little difference between these two ways of deleting nodes in five languages, even after removing up to 5% of the nodes. Thus, unlike transportation networks with vulnerable hubs, language networks can lose many high degree nodes and still maintain connectedness.

**Fig. 2:**
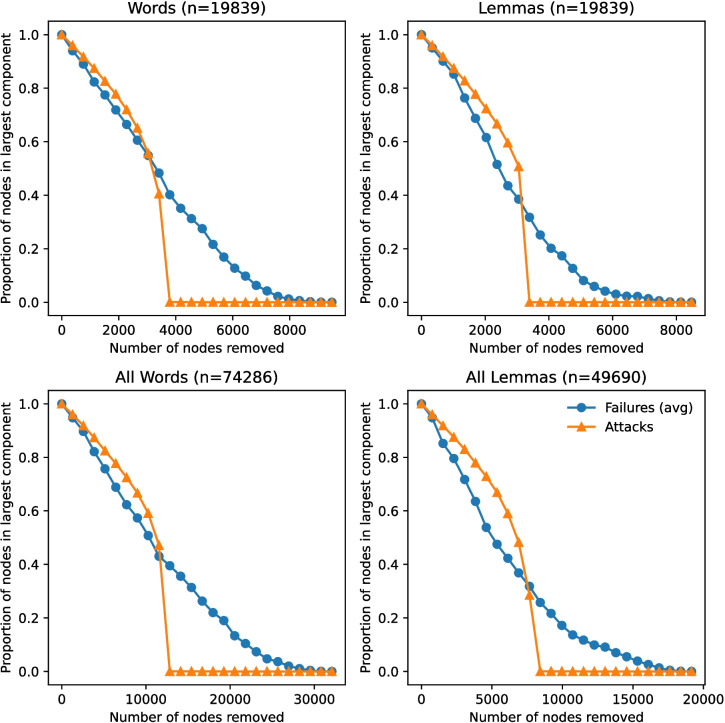
Effect of node removal of random nodes (failures) and by highest degree nodes (attacks) on giant component size

We also investigated the robustness in order to compare phonological networks based on the SUBTLEX-US lexicon and the HML. In particular, we removed nodes at random and by degree in networks with both lemma and word-form representations matched in size with the HML as well as the largest networks. The impact of these two removal processes is shown in Fig. 2 on the proportion of nodes in the giant component. We opt to measure the impact on the giant component, which is a relatively standard technique (Menczer et al., 2020), rather than on the average shortest path length because when a node is deleted, it can either increase or decrease the average shortest path length, which complicates analysis. As shown in all four plots, there is little difference between random node removal (failures) and node removal by descending degree (attacks) in the beginning stages of node removal. In particular, there are only negligible differences between these two processes up until roughly 39% of the giant component nodes are removed, after which the connectedness of this component drops dramatically in attacked networks. All phonological networks, regardless of word representation and size, are also assortative in the sense that they have large and positive correlations in degree of the nodes they are connected to (see Table 5), which further supports their robustness. These findings are consistent with those of Arbesman et al. (2010) and Vitevitch et al. (2023), which also found that language networks are robust to node removal and had assortative mixing by degree.

### Comparison with matched stimuli groups

How do the values of specific measures from the SUBTLEX phonological networks compare to ones derived from other networks? We address this question by comparing network science measures from prior studies and those from a comparable SUBTLEX network. These studies carefully constructed groups of stimuli that differed significantly in network science measure but were matched on other characteristics relevant to the behavioral effects they investigated, such as phonological size and word frequency, so their differences were not predictable from these other variables. Our rationale is that if the SUBTLEX-US words have comparable values, the differences between the two groups should be similar to the differences in these prior studies. The measures used include degree (Arnold et al., 2005; Chan & Vitevitch, 2015; Munson & Solomon, 2004) and clustering coefficient (Chan & Vitevitch, 2009, 2010; Vitevitch et al., 2012). All these studies, except Arnold et al. (2005), used a lemma-based phonological similarity network based on the Hoosier Mental Lexicon. Accordingly, we calculated the same mean values for the two sets using a sized-matched lemma-based network. The rows in Table 7 examine the degree and clustering coefficient values from these studies, and calculate the mean of each group (i.e., high vs. low) and the difference between groups, for both phonological networks. A few stimuli from prior studies could not be compared because they were not present in the SUBTLEX-US corpus, but this was only about 7% of the 387 stimuli words considered.

**Table 7:** Comparison of degree and clustering coefficient for two phonological networks, HML and SUBTLEX-US

		Measures from past studies			Measures from SUBTLEX network		
	*N*	Low (SD)	High (SD)	Diff, %Δ	Low (SD)	High (SD)	Diff, %Δ
Degree							
Arnold et al., 2005	10	7.0 (1.2)	12.6 (4.6)	5.6, 80%	17.4 (5.6)	48.2 (26.1)	30.8, 117%
Chan & Vitevitch, 2015 E1	59	15.93 (2.7)	27.5 (2.0)	11.4, 73%	23.96 (6.8)	35.7(6.8)	11.8, 49%
Munson & Solomon 2004 E2a	34	21.2 (6.3)	23.2 (7.7)	2.0, 10%	29.1 (10.7)	30.0 (10.0)	0.82, 3%
Munson & Solomon 2004 E2b	38	19 (7.0)	24.2 (6.4)	5.2, 27%	26.2 (8.6)	32.5 (8.7)	6.3, 24%
Clustering coefficients							
Chan & Vitevitch, 2009	72	0.25 (0.03)	0.35 (0.04)	0.10, 40%	0.27 (0.05)	0.37 (0.08)	0.09, 34%
Chan & Vitevitch, 2010	53	0.28 (0.05)	0.34 (0.06)	0.07, 23%	0.31 (0.06)	0.37 (0.12)	0.06, 21%
Vitevitch et al., 2012 E1	27	0.22 (0.02)	0.58 (0.12)	0.36, 164%	0.25 (0.04)	0.56 (0.13)	0.31, 122%
Vitevitch et al., 2012 E2	37	0.30 (0.05)	0.53 (0.15)	0.23, 76%	0.27 (0.04)	0.45 (0.18)	0.18, 65%
Vitevitch et al., 2012 E3	30	0.24 (0.03)	0.35 (0.04)	0.11, 47%	0.26 (0.03)	0.37 (0.06)	0.11, 41%

In all nine comparisons, the difference in the SUBTLEX-US network was in the same direction as the HML network. That is, high group means always exceeded low group means. Furthermore, while their absolute means are somewhat different, the magnitude of the change from low to high is comparable. Thus, when a change from low to high is great (e.g., Vitevitch et al., 2012 E2) or small (e.g., Munson & Solomon 2004 E2a), so, too, is the magnitude of the change in the SUBTLEX-US network. However, in a few cases (e.g., Munson & Solomon, 2004 E2a), differences between groups in both networks are inside the standard deviations for the stimuli sets, leaving one to wonder if these groups truly represent a robust contrast. We, therefore, pooled the values for these studies (excluding Arnold et al. (2005)) and calculated how well the actual values are correlated (Table 8). It turns out that degree and clustering coefficient are in general highly correlated between the HML and SUBTLEX-US networks, though this effect drops with low clustering coefficient words.

**Table 8:** Correlations in pooled stimuli

	Degree	Clustering coefficient
Low	0.73	0.45
High	0.70	0.70
All	0.77	0.76

This correlation dip for low-frequency words is not an effect of word length because all words examined from these studies (low and high) are monosyllabic, but it could be an effect of the fact that high-frequency words tend to have higher phonotactic probability (see e.g., Zamuner, 2009). High probability portions of high-frequency words may increase the potential that two similar neighbors are also neighbors, whereas this potential may be more random with low-frequency/low-probability words. Therefore, our SUBTLEX-US network may differ more from these other networks in the neighborhood of low-frequency words, and so they are less well correlated.

## Conclusion and accessibility

In order to address resource limitations in network science, we created several phonological similarity networks based on the SUBTLEX-US lexicon (Brysbaert & New, 2009). In particular, we created 17 networks that differed in size and word representation by constructing adjacency lists for them. These adjacency lists were then examined to determine if network size and word representation impacted network characteristics, as well as if the networks based on the SUBTLEX-US lexicon differed in important ways with a network based on the Hoosier Mental Lexicon. While we did find one difference with the size of the HML’s giant component, the comparable SUBTLEX network’s giant component was not unusual cross-linguistically. This difference was rather minor compared with a host of similarities between the HML and SUBTLEX networks. Both networks exhibit small-world properties, similar degree distributions, and robustness to node removal. This common ground was found in all networks, regardless of size or whether lemmas or word forms are used, supporting related findings in Shoemark et al. (2016) and as well as a study that compared the HML network to one based on experimentally elicited phonological associate data (Castro & Vitevitch, 2023). Both networks also exhibited similar effects on network structure after the extraction of a backbone network exposing important words in the network. Finally, we examined differences in specific values of degree and clustering coefficient for experimental items carefully selected to represent contrasts on these measures, and found comparable values, and comparable contrasts between groups, in the SUBTLEX network.

It seems, therefore, that the two networks represent fundamentally the same kinds of graph-theoretic properties, and both data sets can be used in future research. In terms of the practical differences between them, researchers familiar with SUBTLEX-US will know that it contains many odd ‘words’ like *agr* and some misspellings (approximately 2–3% of the data, based on a 200-word step sample), which introduce some noise into the data. Together with the fact that about a third of the transcriptions were produced programmatically and exhibit some variant pronunciations (see Sect. 2), some researchers may wish to stick to using networks based on the HML data set. On the other hand, we feel that we have convincingly shown that this variability does not lead to significant differences in the networks made from the two data sets. These findings parallel those of Brysbaert and New (2009), who showed that frequency norms based on the word-form representations of the SUBTLEX corpus, which also included some odd words, were just as predictive of behavioral patterns as those based on lemma representations. Furthermore, the access to the underlying data of the networks supports a whole host of research endeavors that are not currently possible for the HML data set. For example, the global measure of closeness centrality is relevant to a host of psycholinguistic behaviors (Castro et al., 2020; Goldstein & Vitevitch, 2017; Sudarshan Iyengar et al., 2012), yet it is generally not available from online psycholinguistic repositories. By applying computational methods like those available in Python’s networkX package to the adjacency lists available with this article, researchers can easily generate measures like this, as well as values for measures that are generally available but are not available for all lexical items they wish to employ in their experiments. A network based on the SUBTLEX-US corpus may be a real benefit in this context because it includes both headwords and related inflected word forms (instead of just the lemmas of the HML) and drawing from TV and film media, it can support research on more colloquial words, as for example found in social media platforms. More broadly, the underlying data gives researchers more room to explore language network science, including important properties like community structure and multi-plex networks (in combination with other data sets).

All of the digital resources for this project are available from the GitHub project page: https://github.com/aldo-git-bit/phonological-similarity-networks-SUBTLEX. This page includes adjlists for all 17 networks, Python programs and functions for generating and analyzing the networks, and documentation that gives an introduction to the functions that underlie the programs. In terms of functionality, the adjlists can be accessed in programming environments such as Python or R, which allows validation and extension of the results reported here for entire networks. Researchers can also use the adjlists and special purpose scripts to extract network science measures for specific experimental items relevant to their research. In addition, our GitHub data pipeline can be expanded in a host of ways, including applying it to other English corpora, languages other than English, and other measures of phonological distance (see the README > Extensions section of the GitHub project page).

## Data Availability

All data generated by the project (e.g., adjacency lists), code for generating the phonological similarity networks, and further code for the analysis and use of the networks is available at the GitHub repository given below, which also acknowledges other code components and data from outside sources. https://github.com/aldo-git-bit/phonological-similarity-networks-SUBTLEX

## Footnotes

1 There is a slight difference in node count between our ~ HML node network (*n* = 19,839, based on Nusbaum et al., 1984 data set) and the count of Vitevitch’s (2008) network (*n* = 19,340). We use the larger size to match the official count because of comparisons we make with HML studies below, but the small difference in total size will not affect our outcomes.

2 We were not able to analyze word familiarity because a large percentage of words in SUBTLEX-US are not in public online repositories, but this measure was insignificant in Vitevitch and Sale’s (2023) analysis so it is less important to our comparison.

## The SUBTLEX-US phonological similarity network and sub-networks are based on words of the lexicon of this corpus. That is, the nodes are words, and two nodes are linked by an undirected and unweighted link if they differ in the deletion, addition, or substitution of a single phoneme. We use the standard network science measures below.

Closeness centrality: a centrality measure of a node; the sum of the distances (i.e., path length) between said node and all others in a network
Clustering coefficient: of a node, a value between 0 and 1 that describes the fraction of pairs of the said node’s neighbors that are linked to each other; average clustering coefficient is simply the average value for all nodes in the network
Community: a group of nodes more densely connected to each other than nodes outside the group (though the conceptions of density and connectedness vary across formal definitions)
Degree: a centrality measure of a node; the number of neighbors (see also ‘phonological neighborhood density’, cf., density, given below); average degree is the mean degree of all words in a network; max degree is the highest degree value of a node in a network
Degree assortativity (mixing pattern): correlation in degree of connected nodes; in “assortative” networks, high-degree nodes are linked to other high-degree nodes, and low degree with other low-degree nodes; “dissortative” networks have the opposite pattern (low degree correlation of connected nodes)
Density: of a network, a value between 0 and 1 that describes the fraction of possible links that actually exist in a network
Diameter: the longest shortest path between any two nodes in a network
Giant component: the largest connected component of a network
Heterogeneity parameter *k*: a measure of the breadth of a degree distribution in a network, defined as the ratio of the average squared degree over the square of the average degree in a network
Lexical hermit: a node in a lexical network that has no neighbors
Lexical island: a connected component that is not the giant component
Shortest path length: of a node to another node, fewest number of links connecting two nodes (shortest path selected when there are multiple paths between two nodes); average shortest path length is the mean shortest path length of all nodes in a network

## References

[CR1] Alstott, J., Bullmore, E., & Plenz, D. (2014). powerlaw: A Python package for analysis of heavy-tailed distributions. *PLoS ONE,**9*(1), e85777. 24489671 10.1371/journal.pone.0085777 PMC3906378

[CR2] Arbesman, S., Strogatz, S. H., & Vitevitch, M. S. (2010). The structure of phonological networks across multiple languages. *International Journal of Bifurcation and Chaos,**20*, 679–685.

[CR3] Arnold, H. S., Conture, E. G., & Ohde, R. N. (2005). Phonological neighborhood density in picture naming of young children who stutter: Preliminary study. *Journal of Fluency Disorders,**30*, 125–148. 15949541 10.1016/j.jfludis.2005.01.001

[CR4] Balota, D. A., Yap, M. J., Cortese, M. J., Hutchinson, K. A., Kessler, B., Loftis, B., Neely, J. H., Nelson, D. L., Simpson, G. B., & Treiman, R. (2007). The English Lexicon Project. *Behavior Research Methods,**39*, 445–459. 17958156 10.3758/bf03193014

[CR5] Brysbaert, M., & New, B. (2009). Moving beyond Kučera and Francis: A critical evaluation of current word frequency normals and the introduction of a new and improved word frequency measure for American English. *Behavior Research Methods,**41*, 977–990. 19897807 10.3758/BRM.41.4.977

[CR6] Brysbaert, M., New, B., & Keuleers, E. (2012). Adding part-of-speech information to the SUBTLEX-US word frequencies. *Behavior Research Methods,**44*, 991–997. 22396136 10.3758/s13428-012-0190-4

[CR7] Brysbaert, M., Warriner, A. B., & Kuperman, V. (2014). Concreteness ratings for 40 thousand generally known English word lemmas. *Behavior Research Methods,**46*, 904–911. 24142837 10.3758/s13428-013-0403-5

[CR8] Castro, N., Stella, M., & Siew, C. S. Q. (2020). Quantifying the interplay of semantics and phonology during failures of word retrieval by people with aphasia using a multiplex lexical network. *Cognitive Science,**44*, e12881. 32893389 10.1111/cogs.12881

[CR9] Castro, N., & Vitevitch, M. S. (2023). Using network science and psycholinguistic megastudies to examine the dimensions of phonological similarity. *Language and Speech,**66*(1), 143–174. 35586894 10.1177/00238309221095455

[CR10] Chan, K. Y., & Vitevitch, M. S. (2009). The influence of the phonological neighborhood clustering coefficient on spoken word recognition. *Journal of Experimental Psychology: Human Perception and Performance,**35*, 1934–1949. 19968444 10.1037/a0016902 PMC2791911

[CR11] Chan, K. Y., & Vitevitch, M. S. (2010). Network structure influences speech production. *Cognitive Science,**34*, 685–697. 21564230 10.1111/j.1551-6709.2010.01100.x

[CR12] Chan, K. Y., & Vitevitch, M. S. (2015). The influence of neighborhood density on the recognition of Spanish-accented words. *Journal of Experimental Psychology: Human Perception and Performance,**41*, 69–85. 25485666 10.1037/a0038347

[CR13] Cosgrove, A. L., Kenett, Y. N., Beaty, R. E., & Diaz, M. T. (2021). Quantifying flexibility in thought: The resiliency of semantic networks differs across the lifespan. *Cognition,**211*, 104631. 33639378 10.1016/j.cognition.2021.104631 PMC8058279

[CR14] Dell, G. S., & Gordon, J. K. (2003). Neighbors in the lexicon: Friends or foes? In N. O. Schiller & A. S. Meyer (Eds.), *Phonetics and phonology in language comprehension and production, differences and similarities* (pp. 9–37). Mouton de Gruyter.

[CR15] Fourtassi, A., Bian, Y., & Frank, M. C. (2020). The growth of children’s semantic and phonological networks: Insight from 10 languages. *Cognitive Science,**44*, e12847. 32621305 10.1111/cogs.12847

[CR16] Goldstein, R., & Vitevitch, M. S. (2017). The influence of closeness centrality on lexical processing. *Frontiers in Psychology,**8*, 1683. 29018396 10.3389/fpsyg.2017.01683 PMC5622968

[CR17] Gruenenfelder, T. M., & Pisoni, D. B. (2009). The lexical restructuring hypothesis and graph theoretic analyses of networks based on random lexicons. *Journal of Speech, Language, and Hearing Research,**52*, 596–609. 10.1044/1092-4388(2009/08-0004) 19380607 10.1044/1092-4388(2009/08-0004) PMC2947148

[CR18] Hagberg, A., Swart, P. J., & Schult, D. A. (2008). Exploring network structure, dynamics, and function using NetworkX. In G. Varoquaux, T. Vaught, & J. Millman (Eds.), *Proceedings of the 7th Python in Science Conference (SciPy2008)* (pp. 11–15).

[CR19] Humphries, M. D., & Gurney, K. (2008). Network ‘small-world-ness’: A quantitative method for determining canonical network equivalence. *PLoS ONE,**3*(4), e0002051. 18446219 10.1371/journal.pone.0002051 PMC2323569

[CR20] Jones, S. D., & Brandt, S. (2020). Density and distinctiveness in early word learning: Evidence from neural network simulations. *Cognitive Science,**44*, e12812. 31960501 10.1111/cogs.12812

[CR21] Klein, G., Kim, Y., Deng, Y., Senellart, J., & Rush, A. (2017). OpenNMT: Open-Source Toolkit for Neural Machine Translation. In *Proceedings of ACL 2017, System Demonstrations* (pp. 67–72). Association for Computational Linguistics.

[CR22] Kovács, L., Bóta, A., Hajdu, L., & Krész, M. (2021). Networks in the mind–what communities reveal about the structure of the lexicon. *Open Linguistics,**7*(1), 181–199.

[CR23] Luce, P. A., & Pisoni, D. B. (1998). Recognizing spoken words: The neighborhood activation model. *Ear and Hearing,**19*, 1–36. 9504270 10.1097/00003446-199802000-00001 PMC3467695

[CR24] Marian, V., Bartolotti, J., Chabal, S., & Shook, A. (2012). Clearpond: Cross-linguistic easy-access resource for phonological and orthographic neighborhood densities. *PLoS ONE,**7*, e43230. 10.1371/journal.pone.0043230 22916227 10.1371/journal.pone.0043230 PMC3423352

[CR25] Menczer, F., Fortunato, S., & Davis, C. A. (2020). *A first course in network science*. Cambridge University Press.

[CR26] Mphilli, Krawiec-Thayer, M. P., Neri, V., T-Cool, & Benetti, B. (2019). *Converts English text to IPA notation*. In https://github.com/mphilli/English-to-IPA/tree/a17c83eadddfd5888a1078b5632860cf474a5c2d

[CR27] Munson, B., & Solomon, N. P. (2004). The effect of phonological neighborhood density on vowel articulation. *Journal of Speech, Language, and Hearing Research,**47*, 1048–1058. 15605431 10.1044/1092-4388(2004/078) PMC4336539

[CR28] Neal, Z. P. (2022). backbone: An R package to extract network backbones. *PLoS ONE,**17*(5), e0269137. 35639738 10.1371/journal.pone.0269137 PMC9154188

[CR29] Nusbaum, H. C., Pisoni, D. B., & Davis, C. K. (1984). Sizing up the Hoosier Mental Lexicon: Measuring the familiarity of 20,000 words. In I. University (Ed.), *Research on Speech Perception Progress Report No. 10*.

[CR30] Ryan, Z. (2020). *English to IPA translation using a neural network*. In https://github.com/LonelyRider-cs/LING4100_project

[CR31] Satuluri, V., Parthasarathy, S., & Ruan, Y. (2011). Local graph sparsification for scalable clustering. Proceedings of the 2011 ACM SIGMOD International Conference on Management of data,

[CR32] Shoemark, P., Goldwater, S., Kirby, J., & Sarkar, R. (2016). Towards robust cross-linguistic comparisons of phonological networks. In *Proceedings of the 14th Association for Computational Linguistics SIGNORPHON workshop on computational research in phonetics, phonology, and morphology* (pp. 110–120).

[CR33] Siew, C. S. Q. (2013). Community structure in the phonological network. *Frontiers in Psychology,**4*, 553. 23986735 10.3389/fpsyg.2013.00553 PMC3753538

[CR34] Siew, C. S. Q., & Vitevitch, M. S. (2016). Spoken word recognition and serial recall of words from components in the phonological network. *Journal of Experimental Psychology: Learning, Memory and Cognition,**42*, 394–410. 10.1037/xlm0000139 26301962 10.1037/xlm0000139

[CR35] Siew, C. S. Q., & Vitevitch, M. S. (2019). The phonographic language network: Using network science to investigate the phonological and orthographic similarity structure of language. *Journal of Experimental Psychology: General,**148*, 475–500. 10.1037/xge0000575 30802126 10.1037/xge0000575

[CR36] Stella, M. (2019). Modelling early word acquisition through multiplex lexical networks and machine learning. *Big Data and Cognitive Computing*,* 3* bdcc3010010.

[CR37] Sudarshan Iyengar, S., Veni Madhavan, C., Zweig, K. A., & Natarajan, A. (2012). Understanding human navigation using network analysis. *Topics in Cognitive Science,**4*(1), 121–134. 22253185 10.1111/j.1756-8765.2011.01178.x

[CR38] Tiv, M., Gullifer, J., Feng, R., & Titone, D. (2020). Using network science to map what Montéal bilinguals talk about across languages and communicative contexts. *Journal of Neurolinguistics*. 10.1016/j.jneuroling.2020.100913 PMC7473004 32905520

[CR39] Turnbull, R. (2021). Graph-theoretic properties of the class of phonological neighborhood networks. In *Proceedings of the workshop on cognitive modeling and computational linguistics* (pp. 233–240). Association for Computational Linguistics.

[CR40] Vitevitch, M. S. (1997). The neighborhood characteristics of malapropisms. *Language and Speech,**40*, 211–228. 9509578 10.1177/002383099704000301

[CR41] Vitevitch, M. S. (2002). Naturalistic and experimental analyses of word frequency and neighborhood density effects in slips of ear. *Language and Speech,**45*, 407–434. 12866911 10.1177/00238309020450040501 PMC2542844

[CR42] Vitevitch, M. S. (2008). What can graph theory tell us about word learning and lexical retrieval? *Journal of Speech, Language, and Hearing Research,**51*, 408–422. 18367686 10.1044/1092-4388(2008/030) PMC2535910

[CR43] Vitevitch, M. S., & Castro, N. (2015). Using network science in the language sciences and clinic. *International Journal of Speech-Language Pathology,**17*, 13–25. 25539473 10.3109/17549507.2014.987819 PMC5609822

[CR44] Vitevitch, M. S., Castro, N., Mullin, G. J. D., & Kulphongpatana, Z. (2023). The Resilience of the Phonological Network May Have Implications for Developmental and Acquired Disorders. *Brain Sciences,**13*, 1–26. 10.3390/brainsci13020188 PMC9954478 36831731

[CR45] Vitevitch, M. S., Chan, K. Y., & Goldstein, R. (2014). Insights into failed lexical retrieval from network science. *Cognitive Psychology,**68*, 1–32. 24269488 10.1016/j.cogpsych.2013.10.002 PMC3891304

[CR46] Vitevitch, M. S., Chan, K. Y., & Roodenrys, S. (2012). Complex network structure influences processing in long-term and short-term memory. *Journal of Memory and Language,**67*, 30–44. 22745522 10.1016/j.jml.2012.02.008 PMC3381451

[CR47] Vitevitch, M. S., & Sale, M. (2023). Identifying the phonological backbone in the mental lexicon. *PLoS ONE,**18*(6), e0287197. 37352192 10.1371/journal.pone.0287197 PMC10289336

[CR48] Watts, D. J., & Strogatz, S. H. (1998). Collective dynamics of ‘small-world ‘networks. *Nature,**393*(6684), 440–442. 9623998 10.1038/30918

[CR49] Weide, R. (2014). *The Carnegie Mellon pronouncing dictionary [cmudict.0.7b]*. http://www.speech.cs.cmu.edu/cgi-bin/cmudict

[CR50] Zamuner, T. S. (2009). Phonotactic probabilities at the onset of language development: Speech production and word position. *Journal of Speech, Language, and Hearing Research,**52*, 49–60. 18723600 10.1044/1092-4388(2008/07-0138)

